# Newborn Screening for X-Linked Adrenoleukodystrophy

**DOI:** 10.3390/ijns2040015

**Published:** 2016-12-06

**Authors:** Ann B. Moser, Richard O. Jones, Walter C. Hubbard, Silvia Tortorelli, Joseph J. Orsini, Michele Caggana, Beth H. Vogel, Gerald V. Raymond

**Affiliations:** 1 Kennedy Krieger Institute, Baltimore, MD 21205, USA;; 2 Division of Clinical Pharmacology, Johns Hopkins University School of Medicine, Baltimore, MD 21287, USA (retired);; 3 Biochemical Genetics Laboratory, Mayo Clinic College of Medicine, Rochester, MN 55905, USA;; 4 Newborn Screening Program, Wadsworth Center, New York State Department of Health, Albany, NY 12201, USA;; 5 Department of Neurology, University of Minnesota Medical Center, Minneapolis, MN 55455, USA;

**Keywords:** newborn screening, X-linked adrenoleukodystrophy, adrenal insufficiency, hematopoietic cell transplantation, C26:0 lysophosphatidyl choline, dried blood spot, tandem mass spectrometry (LC-MS/MS), peroxisomal disorders

## Abstract

Early diagnosis of males with X-linked adrenoleukodystrophy (X-ALD) is essential for preventing loss of life due to adrenal insufficiency and for timely therapy of the childhood cerebral form of X-ALD with hematopoietic cell transplantation. This article describes X-ALD, the current therapies, the history of the development of the newborn screening test, the approval by the Secretary of Health and Human Services for the addition of X-ALD newborn screening to the recommended uniform panel of disorders screened as newborns (RUSP) and the successful implementation of X-ALD newborn screening in the state of New York beginning on 30 December 2013. Follow-up guidelines that have been established in New York are outlined. Based on the success of newborn screening in New York, and early results in Connecticut, where X-ALD newborn screening started in December 2015, and in California, where X-ALD newborn screening began in September 2016, we are confident and hopeful that X-ALD newborn screening will expand to include all US states and to countries that have established neonatal screening programs. The Minster of Health in the Netherlands has approved the addition of X-ALD to the newborn screening program with a start date expected in 2017. The states, such as Massachusetts, Illinois, Minnesota, New Jersey, Florida and Washington, that have legislative approval will commence screening as soon as budgetary resources, testing and follow-up procedures are in place.

## 1. Introduction

X-linked adrenoleukodystrophy, X-ALD, is the most common peroxisomal disorder affecting the adrenal cortex and the central nervous system (brain inflammation and spinal cord/peripheral neuropathy). The two most common forms affecting males are the childhood cerebral X-ALD (CCALD), with the age of onset at two to 10 years (35%), and the adult spinal cord disease adrenomyeloneuropathy (AMN), with the age of onset in the 20s and 30s (60%). About 90% of males also develop adrenal insufficiency. By the age of 60, 80% of female carriers also present with the neurological symptoms of AMN, with a range of symptoms from mild to severe disability. Adrenal insufficiency is rare in females. X-ALD is caused by mutations in the gene ABCD1 which maps to Xq28 and encodes the adrenoleukodystrophy protein (ALDP) which facilitates the transport of very-long-chain fatty acids (VLCFA) into the peroxisome for degradation. ABCD1 mutations lead to an increase in VLCFA in all tissues [1,2]. There are more than 600 known mutations in ABCD1, and there is no genotype/phenotype correlation, even within the same family [3–5]. Through family screening and ascertainment of affected males and females, the overall estimated minimum birth incidence of males and females with X-ALD in the United States was determined to be about one in 17,000 births [6].

Historically the diagnosis of CCALD in boys was established by neuroimaging following early symptoms of attention deficit hyperactivity disorder, ADHD, failure in school, difficulties in understanding language, behavior disturbances, and a decline in handwriting. The characteristic brain MRI pattern found for X-ALD [7,8] led to the biochemical test, the measurement of plasma total lipid VLCFA [9], and to the sequencing of the ABCD1 gene for accurate testing of female relatives who are at risk of being carriers [5]. However, most boys diagnosed with X-ALD after developing neurological symptoms do not respond well to current established therapies.

Current therapies for boys with X-ALD include: Hematopoietic cell transplantation (HCT), if done at the first sign of abnormal brain MRI as measured by a X-ALD MRI (Loes) score of 1 or 2, using a matched related donor or matched cord blood cells, halts the progression of the disease [10,11]. Early results of clinical trials of autologous gene transplantation using Lenti viral vector are promising [12]. Hormone therapy for adrenal insufficiency is well established [13]. Dietary therapy with Lorenzo’s oil which may lower the risk of the CCALD in asymptomatic X-ALD boys aged 1 to 10 years is available [14–16]. X-ALD boys and girls are neurologically normal at birth, and thus there is a “window of opportunity” for these established therapies following early diagnosis through newborn screening.

## 2. Development of the Newborn Screening (NBS) Test for X-ALD

In 2004, at the meeting of the National Advisory Committee for Newborn Screening, Dr. Hugo Moser suggested that X-ALD be added to the list of disorders under consideration for addition to the RUSP in the United States. However, there was no valid test for NBS. Consequently, Dr. Moser recruited a team of researchers, including the technicians and staff of the Peroxisomal Diseases Laboratory at the Kennedy Krieger Institute, Walter Hubbard, an expert in the LC-MSMS analysis of lipids, from the Department of Clinical Pharmacology at Johns Hopkins, and Silvia Tortorelli from the Biochemical Genetics Laboratory at the Mayo Clinic. He obtained funding from several X-ALD support organizations for a pilot study to establish an X-ALD NBS test. In 2006 the team published a preliminary report showing that with the LC-MS/MS assay, C26:0 lysophosphatidyl choline (C26:0LPC) was elevated in postnatal venous dried blood spots (DBS) from X-ALD males compared to normal controls [17]. Validation of the assay was made possible by the custom synthesis of D4-C26:0LPC and other natural VLCFA LPCs by Avanti Polar Lipids and in known positive NBS blood specimens from the California and Michigan state archives retrieved from storage, compared with apparently normal NBS samples from the Centers for Disease Control and Prevention, CDC, and the newborn screening programs of Costa Rica, and the states of California and Maryland [18]. With a grant from the National Institute of Child Health and Human Development, together with the Maryland NBS laboratory and the staff at four local hospitals, the group at the Kennedy Krieger Institute completed a study of 4689 routine newborn blood spots from Maryland, thus demonstrating that NBS for X-ALD is feasible [19]. Other methods including negative-ion LC-MS/MS to reduce the isobaric contaminants of 26:0LPC and other improvements for the high-throughput assay were developed [20,21]. In 2014 the Newborn Screening Quality Assurance Program at the CDCproduced quality control standard blood spots with measured amounts of C24:0 and C26:0LPC and made them available to all laboratories for X-ALD NBS [22]. The Biochemical Genetics Laboratory at the Mayo Clinic developed a high-throughput method and screened 100,000 anonymous NBSs from the California state NBS archives [21]. In 2012, X-ALD families and researchers made an application to the Secretary’s Advisory Committee on Heritable Disorders in Newborns and Children (SACHDNC) to add X-ALD to the RUSP. The application was approved by the SACHDNC on 25 August 2015 and Secretary Burwell signed the letter of approval in February 2016.

## 3. New York is the First State to Add NBS for X-ALD

Following the April 2012 death of their son, Aiden, who had CCALD, the Seeger family drafted Aiden’s Law and lobbied the New York state legislature to add X-ALD NBS. In August 2012, a bill was submitted for the addition of X-ALD to New York State’s NBS panel. The bill was approved by the state Finance Committee in February 2013 and became law in March 2013. On 30 December 2013, the New York state’s NBS laboratory began testing for X-ALD.

NBS for X-ALD in New York State is accomplished using a three-tier algorithm. The first tier is standard MS/MS of C26:0 LPC, followed by measurement of C26:0 LPC using HPLC–MS/MS as the second-tier test [18,21]. All newborns are screened with the first tier and newborns with an out-of-range result are screened with the more specific second tier. If the C26:0 LPC remains elevated on the second tier, then third-tier sequencing of the ABCD1 gene is performed [5] (Table 1).

## 4. Recommended Protocols for a Positive Newborn Screen for X-ALD [23]

The primary physician is notified and a referral is made to genetics for confirmation of the diagnosis, along with genetic counseling for family support services and screening of other family members at risk of X-ALD. For males, it is imperative to implement early in life: (a) serial monitoring by neuroimaging to detect the earliest evidence of progressive cerebral disease; and (b) adrenocortical function testing to detect adrenal insufficiency. Boys with X-ALD should undergo comprehensive neurologic, neuropsychological, neuroradiology, and adrenal function evaluations with subsequent serial monitoring at least every six months during the first decade of life with annual monitoring thereafter. The goals of these recommendations are: (1) to detect cerebral disease early, prior to the development of neuropsychological and/or neurologic signs and symptoms; and (2) to identify adrenal insufficiency, a potentially life-threatening condition, and to treat it appropriately. The X-ALD MRI score of a boy with X-ALD should be independently confirmed by experts in X-ALD prior to recommendation for assessment for HCT. By using such a monitoring strategy, timely and effective HCT will be possible. It is important to note that such a monitoring strategy is necessary since there is currently no test to predict the phenotype in X-ALD.

## 5. Recommended Follow-Up Procedures for Babies with No ABCD1 Mutation [24]

The newborn screening assay of C26:0LPC also detects other conditions of peroxisomal fatty acid oxidation such as the Zellweger spectrum disorders (approximately one in 70,000 births), the less frequent peroxisomal fatty acid oxidation disorders, peroxisomal acyl-CoA oxidase 1 (ACOX1), the multifunctional protein (HSD1B4), and the “contiguous ABCD1 DXS1357E deletion syndrome” (CADDS). In New York, the babies with a second-tier positive screening test who are negative for the ABCD1 mutation are referred to a geneticist where additional postnatal tests are done to elucidate the primary defect. Once the biochemical or genetic diagnosis is established, the family is referred for genetic counseling.

## 6. Discussion

The test for newborn screening for X-ALD is diagnostic and has been implemented in the states of New York, Connecticut and California with a birth incidence of positives for X-ALD of one in 14,700 in New York, close to the rate of one in 17,000 predicted by extended family screening. Follow-up procedures are in place so that the available therapies of adrenal hormone replacement and timely referral to HCT are made. Family counseling has also identified other male siblings and relatives with X-ALD, in some cases after years of searching for a diagnosis. For babies identified with the Zellweger spectrum disorders, the diagnostic odyssey is avoided and the family is referred to a geneticist for appropriate care and counseling.

X-ALD newborn screening, having been added to the RUSP, will likely expand to all states and many countries within the next five years, thus improving the clinical outcome of hundreds of X-ALD babies and their X-ALD relatives.

## Figures and Tables

**Table 1.** Data: newborn screening for X-linked adrenoleukodystrophy.

From the New York State, Newborn Screening Program, Wadsworth Center (Dates: 30 December 2013 to 31 August 2016)
Approximate number of newborns screened: 630,000
Total screened positive in first and second tier test: 53
Total ABCD1 mutations identified in third tier test:
10—No mutation
20 males with mutation
22 females/one XXY male with mutation
